# Characterization of Human Group 9 Innate Lymphoid Cells in Response to Allergen Immunotherapy in Patients With Allergic Rhinitis

**DOI:** 10.1111/all.70202

**Published:** 2025-12-26

**Authors:** Ya‐Qi Peng, Te Zhang, Xiao‐Qing Liu, Ismail Ogulur, Qi Sun, Beate Ruckert, Bi‐Xin He, De‐Hua Chen, Hideaki Morita, Hui‐Jing Ye, Qing‐Ling Fu, Cezmi A. Akdis

**Affiliations:** ^1^ Swiss Institute of Allergy and Asthma Research (SIAF) University of Zurich Davos Switzerland; ^2^ Department of Otorhinolaryngology, Department of Allergy, The First Affiliated Hospital of Sun Yat‐sen University Guangzhou China; ^3^ Department of Otolaryngology‐Head and Neck Surgery, Guangdong Provincial People's Hospital (Guangdong Academy of Medical Sciences) Southern Medical University Guangzhou China; ^4^ Christine Kühne‐Center for Allergy Research and Education Davos Switzerland; ^5^ State Key Laboratory of Ophthalmology Zhongshan Ophthalmic Center, Sun Yat‐Sen University, Guangdong Provincial Key Laboratory of Ophthalmology and Visual Science Guangzhou China; ^6^ Department of Allergy and Clinical Immunology National Research Institute for Child Health and Development Tokyo Japan

**Keywords:** IL‐9, innate lymphoid cells, subcutaneous immunotherapy, transforming growth factor beta

## Abstract

**Background:**

Innate lymphoid cells (ILCs) are classically divided into three groups: ILC1, ILC2, and ILC3 to reflect their functional analogy to Th1, Th2, and Th17. There is also an IL‐9 single‐producing T cell subset, namely the Th9 cell, which plays a dominant role in the onset of allergic diseases compared with traditional Th2 cells. Although a corresponding cell subset of ILCs to different Th cell subsets exists, so far the counterpart of Th9 cells in ILCs has not been reported.

**Objective:**

In this study, we aimed to report the existence of group 9 innate lymphoid cells (ILC9s) and characterize them in allergic rhinitis (AR) and in response to allergen immunotherapy.

**Methods:**

The expressions and characterization of ILC9s were investigated in purified ILCs cultures, PBMCs from patients with AR and responder subcutaneous immunotherapy (SCIT) patients by flow cytometry, scRNA‐seq transcriptome, qRT‐PCR, siRNA knockdown, and immunofluorescence staining.

**Results:**

IL‐9‐expressing cells were observed in the nasal mucosa of patients with AR without the co‐expression of IL‐5 and IL‐13. IL‐4 and TGF‐β induce IL‐9 secretion by human ILCs. scRNA‐seq of whole ILCs defines an H1R^+^OX‐40L^−^ILC subset as ILC9 expressing high levels of IL‐9 and low levels of the ILC2 transcription factor GATA3. Instead, this new ILC9 subset displays Bach2 as a transcription factor, and IL‐9 expression decreases after siRNA inhibition of Bach2. Histamine is an important regulator of ILC9 because ILC9 production increases in response to histamine, and IL‐9 levels in ILCs positively correlate with the expression of histamine 1R. An up‐regulation of PPARγ was observed in ILCs in response to IL‐4 and TGF‐β, and ILC9 differentiation was suppressed by the PPARγ antagonist.

**Conclusion:**

ILC9s are highly expressed in the nasal mucosa and PBMCs of patients with AR and were decreased in response to house dust mite–SCIT. Our study sheds light on the newly discovered ILC9 subset and demonstrates a potential target in allergen immunotherapy.

## Graphical abstract

We aimed to report the existence and profile of group 9 innate lymphoid cells (ILC9s). Overall, ILC9 proportions in AR patients show differences compared to healthy subjects and responder SCIT patients. IL‐9 expression decreases after siRNA inhibition of Bach2 suggesting Bach2 could display as a transcription factor of ILC9. AR, allergic rhinitis; Bach2, BTB and CNC homolog 2; HDM, house dust mite; H1R, histamine 1 receptor; IF, immunofluorescence microscopy; ILC, innate lymphoid cell; IL, interleukin; OX40L, tumor necrosis factor ligand superfamily member 4; SCIT, subcutaneous immunotherapy; scRNA‐seq, single‐cell RNA sequencing; siRNA, small interfering RNA; TGF‐β, transforming growth factor‐β.

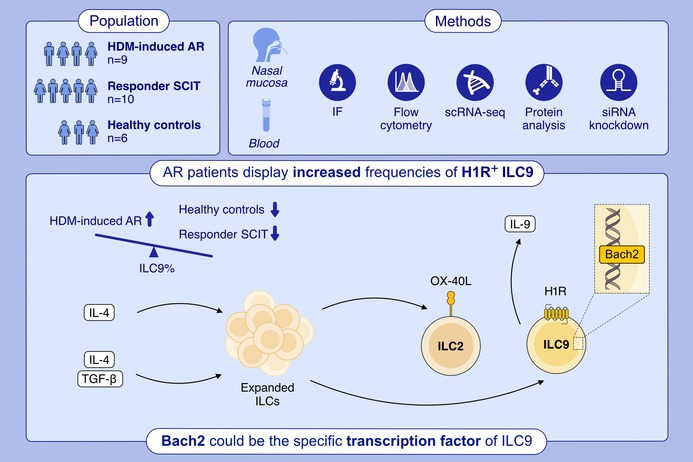

## 1 Introduction

In the past decade, group 2 innate lymphoid cells (ILC2s) attracted much attention in allergies, given their rapid activation by signals deriving from epithelial cells (ECs) [1, 2]. In addition to the typical IL‐5 and IL‐13, some ILC2s secrete IL‐9. IL‐9 is a critical mediator of allergic airway inflammation. It promotes the survival of eosinophils and DCs, thereby facilitating Th2 differentiation, and it can also act on lung macrophages to propagate type‐2 responses [3, 4]. Importantly, IL‐9 functions as a potent amplifier of ILC2 responses by sustaining their survival, driving their accumulation in allergic inflammation, and reinforcing mast cell–ILC2 feedback circuits [5, 6, 7]. IL‐9 is identified as the hallmark cytokine defining T helper 9 (Th9) cells, which was first reported in 1994, production of IL‐9 in the absence of other TH2 or TH1 cytokines stimulated with IL‐4 and TGF‐β [8].

Single‐cell RNA sequencing (scRNA‐seq) has revealed that Th9 cells are the most responsible subset in maintaining the development of asthma along with AR, and in allergic airway recall responses, resident memory CD4^+^ T cells producing IL‐9 have been shown to be essential [9, 10]. Depending on their transcriptional and functional profile, ILCs could mirror the features of the helper T cell subsets. Considering the high plasticity of helper ILCs [11, 12], it is worthy of investigating whether there is an IL‐9‐dominant ILC subset.

To address these questions in a hypothesis‐free manner, we performed an unbiased scRNA‐seq analysis of ILCs stimulated with IL‐4 and TGF‐β, conditions that favor IL‐9 induction [13]. The relatively strong stimulation makes it possible to retain a sufficient number of IL‐9^+^ILCs for high‐throughput single‐cell transcriptomic analysis. Here, our study revealed a distinct subset of ILCs with preferentially higher expression of IL‐9 compared to ILC2, namely ILC9. Although, PU.1 and IRF4 have been reported to be required for the development of Th9 cells [3, 13, 14, 15, 16], we didn't find any differences in ILC9s. While sharing some factors like IL‐9, ILC9s differ from Th9 cells in their specific developmental pathways, particularly regarding the key transcription factors PU.1 and IRF4.

The identified surface markers and the transcription factor of ILC9, namely the BTB and CNC homolog 2 (Bach2) uncovered a unique ILC subset, which might play an important role in airway allergic inflammation. Bach2 was discovered to be highly expressed in CD4 and CD8 T cells and played a significant role in T cell differentiation to Th1, Th2 cells, and regulatory T cell (Treg) [17, 18, 19]. Emerging evidence suggested a critical role of Bach2 involved in the Th2 immune response and associated inflammatory diseases. However, the function of Bach2 in IL‐9‐mediated inflammation is still controversial. The binding of Bach2 on the *il9* promoter region has been reported to induce IL‐9 expression, which supported our finding that Bach2 could be the specific transcription factor of ILC9s [20].

Accordingly, the contributions of ILC9 in the pathogenesis of allergic rhinitis (AR) and the disease‐modifying effects of allergen immunotherapy (AIT) are of interest. AR is characterized by nasal mucosal inflammation, which is caused by an imbalance in the immune response to allergens [21, 22]. AIT, the only disease‐modifying therapy for AR, has been shown to rebalance immunity and induce changes in innate lymphoid cells (ILCs), suggesting that ILCs may serve as potential biomarkers of treatment efficacy. During AIT, ILC2, and ILC3 decrease while ILC1 and a newly identified ILC subgroup that produces IL‐10 increase, resulting in a ratio similar to healthy subjects [23, 24]. These together indicated that ILC subtype shifting might be involved in the mechanism of AIT.

Here, we propose a plastic functional subset of ILCs as histamine H1 receptor expressing (H1R)^+^ILC9. Histamine plays a key role in allergic diseases that have long been thought to be mediated by the H1R, and H1‐receptor antagonists—commonly known as antihistamines—have been used to treat allergies for many years. Histamine appears as a strong regulator as ILC9 production increases in response to histamine, and IL‐9 levels in ILCs positively correlate with the expression of H1R. The increased proportion of ILC9 in acute exacerbation of allergic rhinitis and their decrease in response to successful AIT further demonstrate an in vivo functional role.

## 2 Materials and Methods

### 2.1 Subjects

The study was approved by the Ethics Committee of The First Affiliated Hospital, Sun Yat‐sen University, China. For in vitro cross‐sectional study (Figures 5 and 6), patients receiving house dust mite (HDM) SCIT (*n* = 10; Alutard SQ, ALK Hørsholm, Denmark), untreated HDM‐allergic patients (*n* = 9), and healthy controls (HC, *n* = 6) were recruited and completed symptom questionnaires. AR subjects were diagnosed by positive HDM specific IgE and skin prick test (SPT, wheal diameter 3 mm greater than the negative control) with the duration of diseases for over 6 months. The blood of patients receiving SCIT was collected 6–18 months after the initiation of the treatment. Inclusion and exclusion criteria for patients with AR or HC were described in our previous study [25, 26, 27]. None of the AR subjects has used oral or nasal corticosteroid or other treatments (e.g., H1‐antihistamine or immunotherapy) for 6 weeks before the study. There was no comorbidity in AR subjects with asthma. SCIT‐treated patients have received standard immunotherapy for 6 months to 3 years. Baseline characteristics and clinical parameters are given in Table 1.

**TABLE 1:** The characteristics of the subjects' demographics and IgE involved in the study.

Characteristic	Healthy subjects	AR patients	Patients after SCIT
No. of patients	6	9	10
Age (years)	23.83 ± 1.778	23.78 ± 0.969	28.30 ± 2.967
Gender, female/male	5/1	5/4	6/4
SPT, wheal diameter	0 (0%)	9 (100%)	10 (100%)
tIgE concentration (IU/mL)	24.45 ± 6.82	77.71 ± 7.38***	184.7 ± 33.34****
sIgE concentration (IU/mL)			
Der p/Der f	0.077 ± 0.01	88.133 ± 7.49*	20.65 ± 7.321***
Trees	< 0.35	< 0.35	< 0.35
Artemisia/ragweed	< 0.35	< 0.35	< 0.35
Molds	< 0.35	< 0.35	< 0.35
Hair or dander of pets	< 0.35	< 0.35	< 0.35
VAS	1.22 ± 0.32	22.13 ± 2.56**	19.38 ± 5.06**
TNSS	0.375 ± 0.18	6.0 ± 0.58**	3.7 ± 1.07

*Note:* Data are presented as mean ± SEM. Mann–Whitney *U* tests were used to test for significant differences between groups. All statistically significant differences were defined as *p* values *p < 0.05, **p < 0.01, ***p < 0.001, ****p < 0.0001 compared to healthy subjects. The *p* values are listed as follow: tIgE levels (*p* = 0.0006 HC vs. AR, *p* < 0.0001 HC vs. AR after SCIT), sIgE levels (*p* = 0.011 HC vs. AR, *p* = 0.0004 HC vs. AR after SCIT), VAS (*p* = 0.0027 HC vs. AR, *p* = 0.0056 HC vs. AR after SCIT), VAS (*p* = 0.0023 HC vs. AR, *p* = 0.1882 HC vs. AR after SCIT).

Abbreviations: sIgE, specific immunoglobulin E; SPT, skin prick tests; tIgE, total immunoglobulin E; TNSS, total nasal symptom score; VAS, visual analogue scale.

For ex vivo ILC analyses, human blood buffy coats from “anonymous donors” were from Guangzhou Blood Center, and the exemption of written informed consent was approved. Written informed consent was obtained from every participant. Blood samples from all participants were anonymized and blinded before the laboratory processing. All research laboratory staff were blinded to the treatment groups, and all experiments were performed blinded to the treatment groups.

### 2.2 Human Nasal Tissue

**FIGURE 1:**
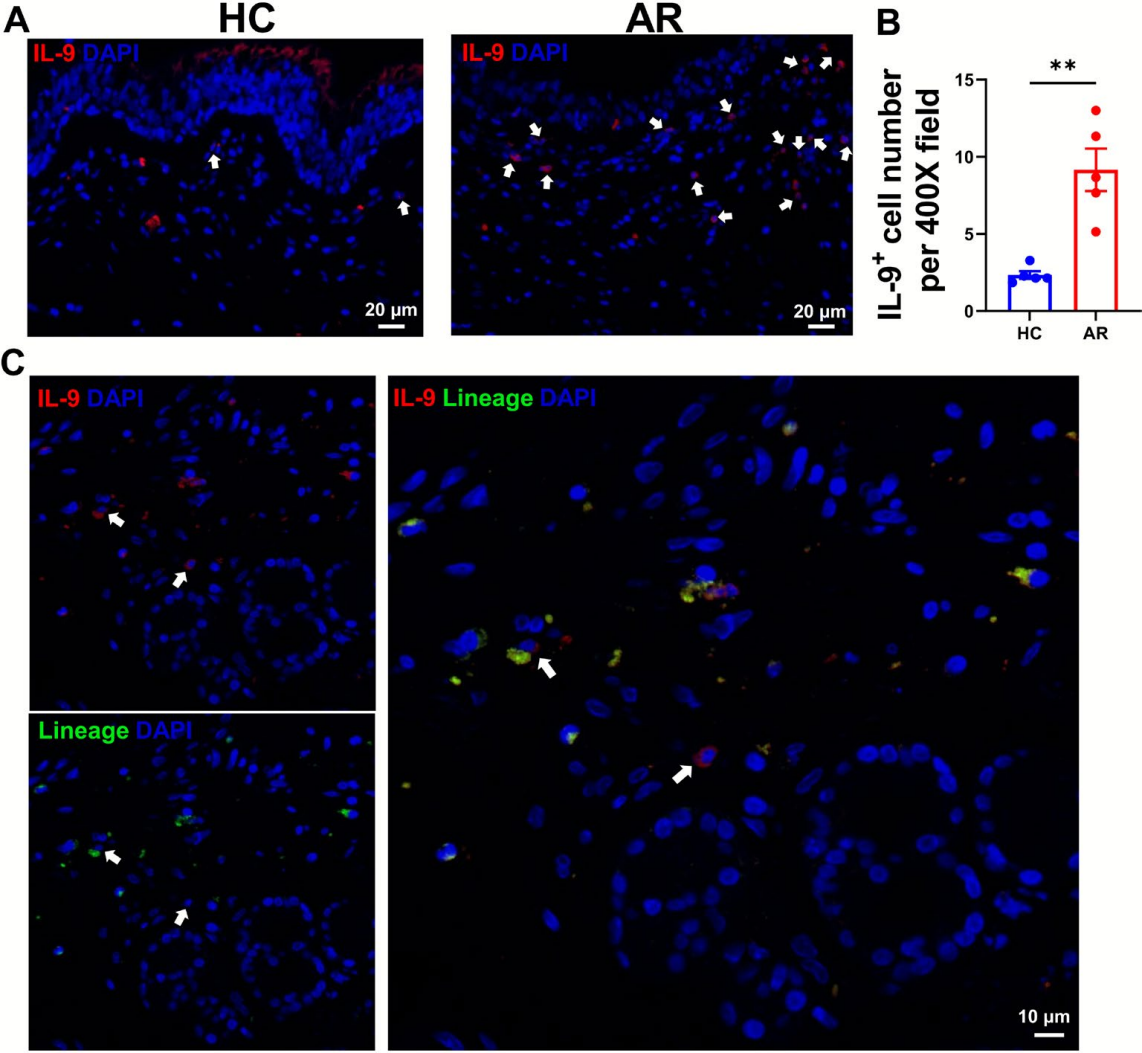
Higher levels of IL‐9‐expressing cells and the existence of ILC9 in the nasal mucosa of patients with AR. Immunofluorescence staining of human nasal mucosa from nonallergic donors and patients with AR. Lineage (including CD2, CD16, CD19, CD56, CD235a, and FcɛRI, green), IL‐9 (red), and DAPI (blue). (A, B), IL‐9‐expressing cells in nasal mucosa were stained in red. Average numbers of the IL‐9‐expressing cells were calculated in 7400× high‐power fields per subject. (C) ILC9 was defined by staining of Lineage^−^IL‐9^+^ cells in nasal mucosa from patients with AR. IL‐9‐expressing cells or ILC9s were pointed out by white arrows.

Inferior turbinate mucosal samples (Figure 1) were collected from patients with HDM‐allergic AR and nonallergic patients with nasal septal deviation undergoing septoplasty. The study was approved by The Ethics Committee of Tongji Hospital, Tongji Medical College, Huazhong University of Science and Technology, and conducted with written informed consent from every patient as those in our previous study [28]. Human palatine tonsil tissue samples (Figure S1) were obtained from hospitals in Davos and Chur, Switzerland. Patients underwent elective tonsillectomy because of hypertrophic and obstructive tonsils.

### 2.3 Expansion of Human ILC Lines and ILC9 Priming

Human peripheral blood mononuclear cells (PBMCs) of the buffy coat (Redcross Switzerland) were isolated using gradient centrifugation. After depletion of CD3^+^, CD16^+^, CD19^+^, and CD14^+^ cells by autoMACS (Miltenyi Biotec, Bergisch Gladbach, Germany), Live^+^CD45^+^Lin^−^CD127^+^ cells were sorted (Figure S2A) with a FACSAria II (BD Biosciences, Franklin Lakes, NJ, USA). Purified ILCs were expanded in Yssel medium supplemented with 1% human AB serum together with irradiated allogeneic PBMCs and 100 U/mL recombinant human IL‐2 (Proleukin; Novartis, Basel, Switzerland) for 3 weeks. The typical fold expansion after 3 weeks was 100–300. For human ILC9‐priming, expanded ILCs were seeded 2 × 10^5^ cells per 96‐well and IL‐2 (100 U/mL), IL‐4 (20 ng/mL) and TGF‐β (3 ng/mL, both from R&D Systems, Minneapolis, MN, USA). From the initiation of cell culture until the time point of analysis, the culture medium was supplemented with the indicated cytokines every other day. Cells and supernatants were collected at different time points for analysis by flow cytometry or Meso Scale Discovery (MSD) assays (Rockville, Maryland, USA).

### 2.4 Flow Cytometric Analysis

Cell‐surface protein and intracellular cytokines and transcription factors were stained with specific antibodies: FITC‐conjugated lineage cocktail: anti‐CD1a (HI149), anti‐CD3 (OKT3), anti‐CD11c (3.9), anti‐CD14 (HCD14), anti‐CD16 (3G8), anti‐CD19 (HIB19), anti‐CD34 (581), anti‐CD94 (DX22), anti‐CD123 (6H6), anti‐CD303 (201A), anti–T‐cell receptor (TCR) αβ (IP26), anti‐TCRγδ (B1), and anti‐FcεRIa (AER‐37); PE‐Cy7‐conjugated anti‐CD127 (A019D5); Brilliant Violet 510‐conjugated anti‐CD45 (HI30); PerCP‐Cy5.5‐conjugated anti‐CD161 (HP‐3G10); CCR9; Alexa Fluor 647‐conjugated anti‐IL‐9 (MH9A4); Brilliant Violet 421‐conjugated anti‐IL‐13 (JES10‐5A2); Alexa Fluor 488‐conjugated anti‐PPARγ (polyclonal, all from BioLegend, San Diego, CA, USA); PE‐conjugated antihistamine H1R (480054); BV711‐conjugated anti‐OX40 Ligand (CD252, ik‐1, both from R&D Systems, Minneapolis, MN, USA). Blank control and fluorescence minus one (FMO) staining control for OX‐40 L and H1R were shown in Figure S2B,C. For intracellular cytokine detection, cells were stimulated for 6 h with 20 ng/mL PMA plus 1 μg/mL ionomycin in the presence of 2 μg/mL Brefeldin A (all from Sigma‐Aldrich, St Louis, MO, USA) for the final 3 h of culture and subsequently fixed and stained with a Cytofix/Cytoperm Kit (BD Biosciences, Franklin Lakes, NJ, USA). For staining of transcription factors, the Foxp3 Fix/Perm Buffer set (BioLegend, San Diego, CA, USA) was used.

### 2.5 Single‐Cell RNA‐Seq and Downstream Data Analysis

For scRNA‐seq assays (10× Genomics platform), IL‐2, IL‐4, and TGF‐β stimulated ILCs were collected and sorted into sterile ice‐cold 1.5‐mL collection tubes containing 50 μL Yssel medium supplemented with 1% human AB serum (10^3^ ILCs/μL). Functional Genomics Center Zurich (FGCZ) was employed to perform the cDNA generation, library preparation, and Illumina sequencing (500 M reads) processing. The cells have been run through a formal quality and quantity testing and acceptance process (Quality Control) by FGCZ during each step.

Raw data were transferred to the R statistical environment using Seurat package (v3.0.2). Doublet cells were eliminated from downstream analysis. We performed clustering analysis using distinct lists of the most variable genes for ILC clusters—clustering analysis using uniform manifold approximation and projection (UMAP) dimensional reduction algorithm. scRNA‐seq experiment is deposited at OMIX with accession number: OMIX012222. This paper does not report original code.

### 2.6 Correlation Analysis

The expression correlation between genes was analyzed by Markov Affinity‐based Graph Imputation of Cells (MAGIC) [29]. MAGIC is a commonly applied algorithm for denoising high‐dimensional data, using the nearest neighbor graphing and a diffusion operator to restore or “smooth” missing data transcripts. Then, the results of MAGIC were used to calculate the correlation of genes by the Pearson correlation coefficient.

### 2.7 SCENIC Analysis

To calculate the regulon activity scores (RAS) of ILC clusters, we used the SCENIC (Single‐Cell rEgulator Network Inference and Clustering) pipeline with pySCENIC [30]. First, the co‐expression modules between transcription factors and the candidate target genes were inferred using the grn function with the gene regulatory network reconstruction algorithm “grnboost2” selected. Second, the co‐expression module was further pruned by keeping only direct targets of TFs based on motif discovery by RcisTarget. Then, AUCell was used to evaluate each cell's regulon activity score (RAS). Finally, the differentially activated regulons were recognized in a specific ILC cluster by the Wilcoxon test.

### 2.8 Isolation and Culture of PBMCs

Human PBMCs from patients with AR were cultured in RPMI 1640 medium supplemented with 10% heat‐inactivated fetal calf serum, 10,000 U/mL penicillin, and 10 mg/mL streptomycin at 37°C and 5% CO2. In some experiments, PBMCs were stimulated with histamine (10 μM, Macklin, Shanghai, China) for 24 h or pretreated with PPARγ antagonist, GW9662 (30 μM, Selleck, Houston, USA) for 30 min before the IL‐4 and TGF‐β stimulation for 3 days. In some experiments, the selective H1R agonist 2‐Pyridylethylamine dihydrochloride and the H2R agonist Amthamine dihydrobromide (10 μM, both from Tocris Bioscience, Wiesbaden‐Nordenstedt, Germany) were added to the purified ILCs. Then, ILC9 levels were determined via flow cytometry.

### 2.9 siRNA Knockdown

Human PBMCs from patients with AR were pre‐treated with IL‐4 and TGF‐β for 24 h. Then, siRNAs targeting GAPDH, BACH2 (sense, 5′‐GCCCAGGAAAGAUUAUACCUATT‐3′ and antisense, 5′‐UAGGUAUAAUCUUUCCUGGGCTT′), or scramble were added at a concentration of 100 nanomolar. Cells were harvested 48 h later for IL‐9 and Bach2 RNA analysis.

### 2.10 Immunofluorescence

Immunofluorescence staining on human nasal mucosa was performed using the following antibodies: rabbit antihuman IL‐9 (1:100, Abcam, Cambridge, UK) and FITC‐conjugated lineage cocktails (1:50; eBioscience, San Diego, CA, USA) as primary antibodies; CF543‐conjugated goat anti‐rabbit (1:300; Biotium, Fremont, CA, USA) and AF488‐conjugated goat anti‐mouse (1:300; Invitrogen, Waltham, MA, USA) antibodies were used as secondary antibodies. Images were acquired and analyzed using the confocal microscope Zeiss LSM 710 and ZEN 2.3 lite software (Carl Zeiss Microscopy GmbH, Jena, Germany).

### 2.11 RT‐qPCR

Total RNA was isolated from ILCs with an RNeasy Plus Micro Kit (Qiagen, Hilden, Germany) according to the manufacturer's protocol. Reverse transcription of samples was performed with RevertAid RT Kit containing random hexamers (Thermo Fischer Scientific). Quantitative real‐time PCR (qRT‐PCR) was performed using the iQ SYBR Green Supermix (Bio‐Rad Laboratories, Hercules, Calif) on the Bio‐Rad iCycler. The primer sequences used for qRT‐PCR are shown in the Table S1. Data were analyzed using the $2^{-∆∆C_{T}}$ value and presented as fold changes in gene expression after normalization to the internal control, gene expression level [31]. Expression of the target gene was expressed as a fold increase relative to the expression of Glyceraldehyde‐3‐phosphate dehydrogenase (GAPDH). The mean value of the replicates for each sample was calculated and expressed as cycle threshold (*C*_t_). The amount of gene expression was then calculated as the difference (Δ*C*_t_) between the *C*_t_ value of the target gene and the *C*_t_ value of GAPDH or EF‐1α. Fold changes in target gene mRNA were determined as $2^{-∆C_{t}}$.

### 2.12 ELISA

The cell supernatants were analyzed by using IL‐9 and IL‐13 ELISA kits (Invitrogen, Waltham, MA, USA) and Meso Scale Discovery (MSD) assays (Rockville, Maryland, USA), according to the manufacturer's instructions.

### 2.13 Statistics

Statistical analyses were performed using GraphPad Prism v9.2 software (GraphPad Software, La Jolla, CA, USA). Kolmogorov–Smirnov test was used to test the normality of all the data. Differences between the two groups were analyzed by the paired or unpaired *t*‐test for the data with normal distribution, and the Wilcoxon matched‐pairs signed‐rank test or Mann–Whitney *U* test was performed to compare the data with abnormal distribution. Three or more groups were compared using a one‐way analysis of variance (ANOVA) with the Bonferroni post hoc test for the data with normal distribution, or the Kruskal–Wallis test for the data with abnormal distribution for repeated measurements. Correlations were analyzed with the Spearman rank test. *p <* 0.05 was considered statistically significant.

## 3 Results

### 3.1 Higher Levels of IL‐9‐Expressing Cells in the Nasal Mucosa of Patients With AR

We first analyzed the expression of IL‐9 in the nasal mucosa of healthy subjects and patients with AR. We found that IL‐9‐expressing cells (median number, 9; range, 5–13 in a 400× high‐power field) were present in nasal tissue from patients with AR, yet those were rarely detected in nasal tissue from healthy subjects (median number, 2; range, 1–3 in a 400× high‐power field) (Figure 1A). These results showed that IL‐9‐secreting cells were significantly higher in AR tissue compared to control tissue (*p <* 0.01, Figure 1B), suggesting that IL‐9 might be involved in the allergic inflammatory responses. To demonstrate the in situ presence of Lineage^−^IL‐9^+^ cells in the nasal mucosa of patients with AR (Figure 1C) and to exclude the ILC2 contamination, we added the channel of GATA3 and detected the Lin^−^GATA3^−^IL‐9^+^ cells in human tonsil tissues (Figure S1A). Additionally, we detected the Lin^−^IL‐5^−^IL‐9^+^cells (Figure S1B) and Lin^−^IL‐13^−^IL‐9^+^ (Figure S1C) in the AR tissue. The data demonstrated non‐lineage committed cells secreting IL‐9 without the co‐expression of type 2 cytokines, which may play a role in the pathogenesis of allergic diseases.

### 3.2 IL‐4 and TGF‐β Promote the Induction of IL‐9 and PPARγ in ILCs

**FIGURE 2:**
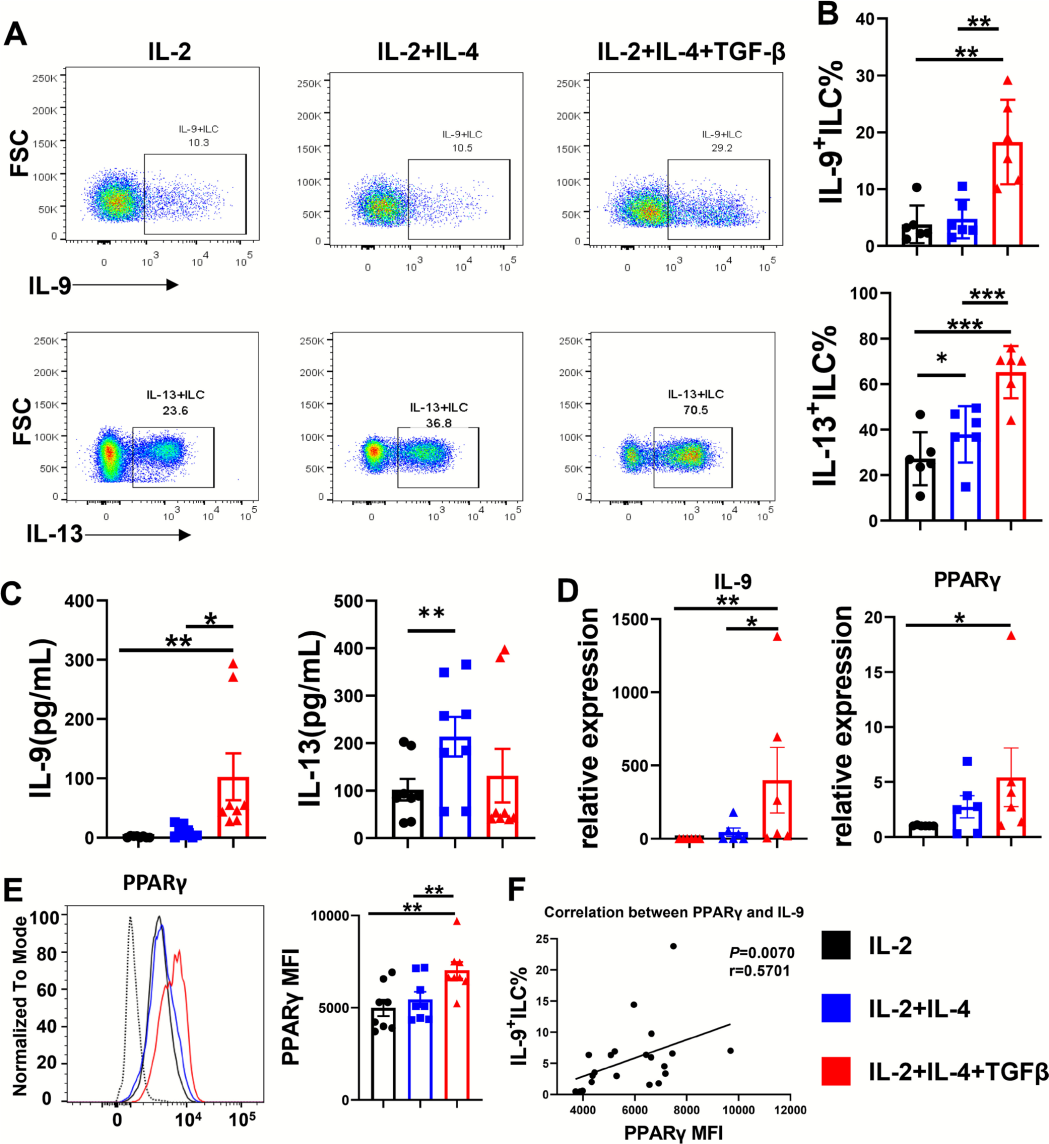
IL‐4 and TGF‐β promote the induction of IL‐9 and PPARγ in ILCs. IL‐9 and IL‐13 production by human ILC in the presence of IL‐2 (100 U/mL), IL‐2 plus IL‐4 (20 ng/mL), or all together with TGF‐β (3 ng/mL) for 7 days: (A) The flow plots of IL‐9^+^ILC and IL‐13^+^ILC. (B) The bar graphs of IL‐9^+^ILC% and IL‐13^+^ILC%. (C) The levels of IL‐9 and IL‐13 in the supernatants. (D) The mRNA levels of IL‐9 and PPARγ. (E) Intranuclear PPARγ were detected by flow cytometry. The bar graph shows the level of PPARγ MFI. (F) The correlation of PPARγ MFI with IL‐9^+^ILC%. The bar graph shows the level. Data are shown as mean ± SEM. *p < 0.05 and **p < 0.01.

ILC subsets are highly plastic, allowing them to respond rapidly to immune environmental changes [11, 32, 33, 34]. Classical ILC2s predominantly produce a dramatic amount of IL‐5 and IL‐13 after activation, but only a little IL‐9 in parallel. To identify the transient ILC9 phenotype, we performed time course analyses of their cytokine expression using different combinations of stimuli. ILCs were purified (Figure S2A) and expanded; the purity of ILCs post‐expansion was routinely greater than 95% (Figure S2B). We found that IL‐4 and TGF‐β induced the IL‐9 secretion by human ILCs in a time‐dependent manner (Figure 2A and Figure S3A). Yet, there was no effect of epidermal growth factor (EGF) and amphiregulin (AREG) on the percentages of IL‐9^+^ILCs regardless of the dosage (Figure S3A), which didn't mirror the characteristics of Th9 cells [35].

Further, we observed that IL‐4 induced IL‐13 (*p <* 0.05) but not IL‐9 production from ILCs. TGF‐β addition rendered, IL‐9^+^ILC percentages more pronounced after 7 days of stimulation (*p <* 0.01, Figure 2B). To confirm this observation, we repeated the examination by IL‐9 detection in the supernatants and RT‐qPCR. We found that TGF‐β together with IL‐4 remarkably enhanced the mRNA synthesis and protein production of IL‐9 (*p <* 0.05) but did not affect the levels of IL‐13 (Figure 2C,D). Further, we sought to detect the profile of cytokines secreted under the stimulus. However, there was no difference in the levels of IFN‐γ, IL‐4, IL‐5, IL‐17A/F, and IL‐10 in the supernatants (Figure S3B). Lineage‐determining transcription factors guided the differentiation of ILC subsets, e.g., GATA‐binding protein 3 (GATA3)‐ILC2, which are also involved in helper T (Th) cell conversions [36, 37, 38]. It has been recently shown that peroxisome proliferator‐activated receptor–gamma (PPARγ) was one of the most highly coexpressed mRNAs together with the transcripts encoding the effector cytokine IL‐9, following TGF‐β stimulation. Next, we attempted to clarify whether PPARγ could contribute to the differentiation of ILC9 in vitro, similar to Th9 cells [39]. IL‐4 alone promoted the levels of GATA3 in ILCs (*p <* 0.05, Figure S3C) without any difference in PPARγ levels. Conversely, the ILC9 priming condition strongly up‐regulated the levels of PPARγ (*p <* 0.01, Figure 2D,E). Intriguingly, the median fluorescence intensities (MFI) of PPARγ were highly correlated with the percentage of IL‐9^+^ILCs (*p* = 0.007, *r* = 0.5701, Figure 2F). Also, PU.1 is important in the development of Th9 cells [13]. Given that, we determined the expression of PU.1 in ILCs under the stimulation of IL‐4 and TGF‐β. There was no significant difference among groups (Figure S3D). It suggested ILC9 could mirror the profile of Th9, yet not the same in the in the aspect of transcription factors.

In conclusion, the data shows that the addition of TGF‐β to ILC priming together with IL‐4 induces the expressions of both IL‐9 and PPARγ in ILCs.

### 3.3 Histamine H1 Receptor‐Expressing ILC9s Identified by Single‐Cell RNAseq in Human Peripheral Blood

The definition of ILC9 using intracellular cytokine restricted our understanding of the functional investigation in terms of access and cell sorting. Recent advances in high‐throughput scRNA‐seq have partially overcome these obstacles. Hence, TGF‐β and IL‐4 primed whole ILC were pooled for droplet‐based scRNA‐seq (10× Genomics platform). With a median of 2177 genes per cell detected in a total of 12,439 cells, unsupervised clustering followed by UMAP visualization revealed 11 clusters (Figure S4A). The top 10 expressed genes in each cluster were displayed in Figure S4B. Clusters 0, 4, and 5 were annotated as ILC2s based on their relatively higher ILC2 gene signature expression e.g., IL5, IL4, and IL1RL1 (Figure S4B). Notably, GATA3, RORA, and IL13 genes, which defined ILC2s, were highly detected throughout the database by scRNA‐seq analysis, likely because of the addition of IL‐4 and the higher sensitivity of this technology. Cluster 1 was characterized by selective expression of IL9 among all the clusters (Figure S4C).

**FIGURE 3:**
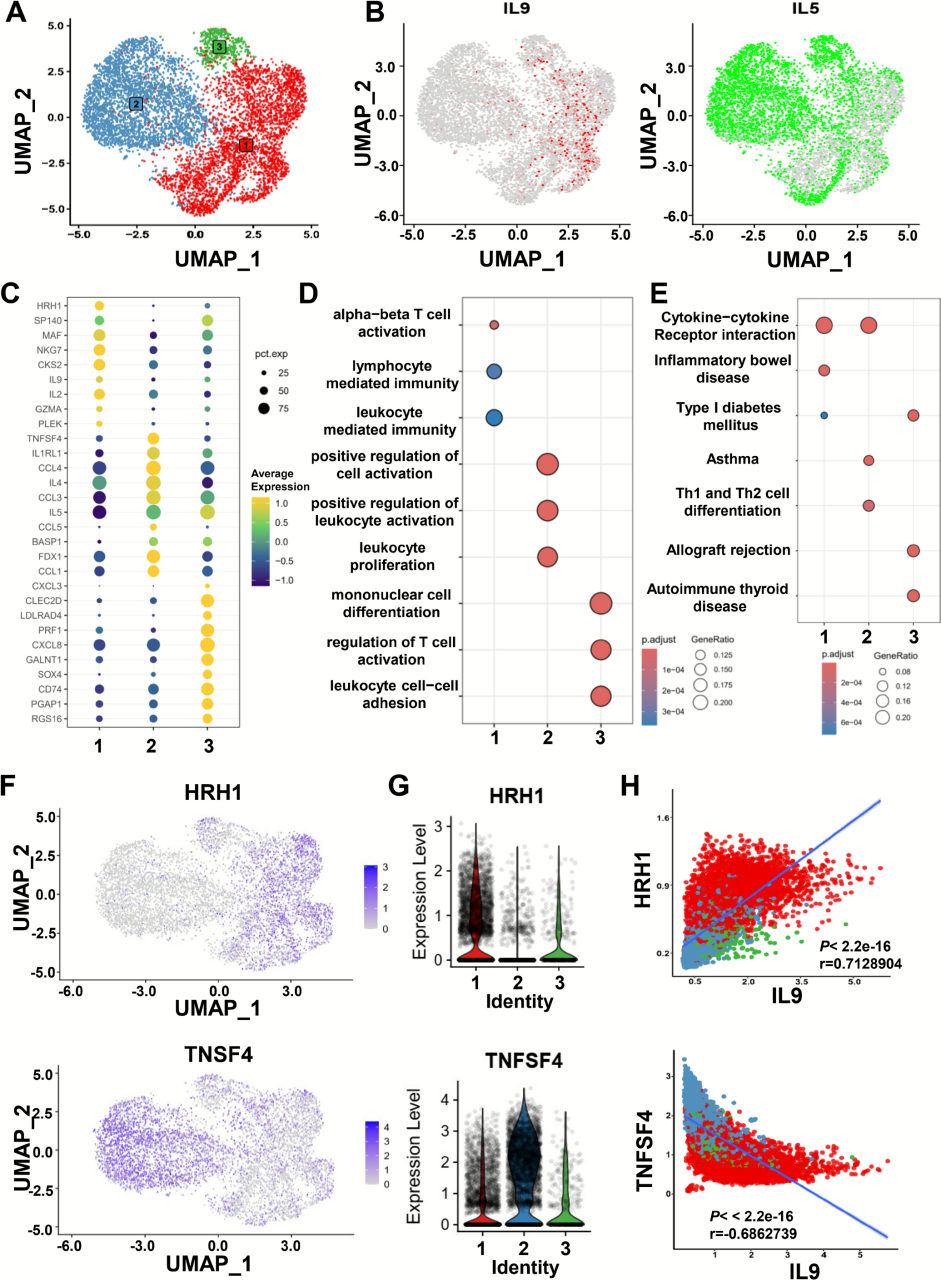
Histamine H1 receptor‐expressing ILC9s identified by single‐cell RNAseq in human peripheral blood. Human ILCs were treated with IL‐2, IL‐4, and TGF‐β for 7 days and then re‐stimulated with PMA and Ion for 4 h before scRNAseq. (A) UMAP visualization of scRNAseq data of selected cells. Colors correspond to phenogram‐based clustering of cell population. (B) The UMAP visualization of IL‐9 and IL‐5 HRH1, TNFSF4. (D) Bubble heatmap highlighted the genes which significantly increased across all the clusters. Percent expressed and average expressed are indicated by dot size and color, respectively. Gene Ontology (GO, E) and Kyoto Encyclopedia of Genes and Genomes (KEGG, F) enrichment analysis of all clusters. (G) The UMAP visualization of HRH1and TNFSF4 G, Expression distribution (violin plots) of HRH1 and TNFSF4 in each cell cluster. (H) The correlation of HRH1 and TNFSF4 with IL‐9.

Given the representativeness of ILC cluster distribution, we also got clusters of ILC1/NK and ILC3, which might distract our analysis regarding allergy‐relevant ILC subsets. To address this issue, we re‐clustered cells only from the ILC2/9 populations (clusters 0, 1, 4, and 5 shown in Figure S4C) and revealed three distinct subclusters (Figure 3A). Newly defined Clusters 2 and 3 were identified as IL‐5^high^IL‐4^high^ILC2 and IL‐5^high^IL‐4^low^ILC2 cells, based on their expressions (Figure 3B and Figure S4D). With the high expression of both IL‐5 and IL‐4, yet a relatively low expression of IL‐9, we named the new cluster 2 as ILC2^super^. As expected, IL‐9^high^ cells were enriched in the new cluster 1, namely ILC9. Further, we intended to investigate the co‐expression of IL‐9^+^ILC9 with other T1/2/3 inflammatory mediators. We found the 29.52% IL‐5^+^IL‐9^+^, 25.48% IL‐17A^+^IL‐9^+^, 9.85% IFN‐γ^+^IL‐9^+^, and 1.1% IL‐10^+^IL‐9^+^ co‐expression (Figure S5A). Overall, there were over 70% IL‐9^+^ILC without the co‐expression of other cytokines.

Next, we sought to identify potential ILC9 surface markers. Single‐cell differential gene expression (DEG) analysis of all the clusters revealed that the histamine H1 receptor (*HRH1*) expression linked to pathogenicity and survival of Th2 cells was significantly enriched in cluster 1 (Figure 3C and Figure S4E). Meanwhile, tumor necrosis factor superfamily member 4 (*TNFSF4*) was highly expressed in the ILC2^super^ cluster, which encodes OX‐40L (Figure 3C and Figure S4E). Gene Ontology (GO, Figure 3D) and Kyoto Encyclopedia of Genes and Genomes (KEGG, Figure 3E) enrichment analysis of all clusters were performed. We found that alpha–beta T cell activation and cytokine–cytokine receptor interaction pathway were enriched in cluster 1. Together, these described the binding of cytokine directly lead to the functional change and activation of T cells. Given the fact that IL‐9 was highly expressed in cluster 1, it indicated that IL‐9^+^ILC could activate T cells by the cytokine secretion, which involved in the inflammatory immunity. Moreover, we showed the UMAP of *HRH1* and *TNFSF4* (Figure 3F), the HRH1^+^TNFSF4^−^ILCs seemed to overlap with IL‐9^+^ILCs, indicating that these cells are responsible for the expression of IL‐9 (Figure 3B,F). Lung OX‐40L^+^ILC2s have been reported to have the capacity to regulate the tissue‐restricted Th2 cell expansion and activation in response to IL‐33 [40, 41]. Cells in Cluster 1 expressed the highest levels of *HRH1*, whereas *TNFSF4* was up‐regulated in Cluster 2 (Figure 3G). We next asked if the varied expressions of high *HRH1* and low *TNFSF4* between clusters are linked to the characteristics to differentiate ILC9. As hypothesized, there was a strong positive correlation between the populations of IL‐9^+^ILCs and HRH1^+^ILCs, and interestingly *TNFSF4* was negatively associated with the expression of *IL‐9* among the overall ILC populations (Figure 3H). Altogether, these data highlighted the differential transcriptomic profile in ILCs and confirmed that ILC9 could be characterized as H1R^+^OX‐40L^−^ILCs as a surface phenotype.

### 3.4 Bach2 Is a Possible Specific Transcription Factor of ILC9s

**FIGURE 4:**
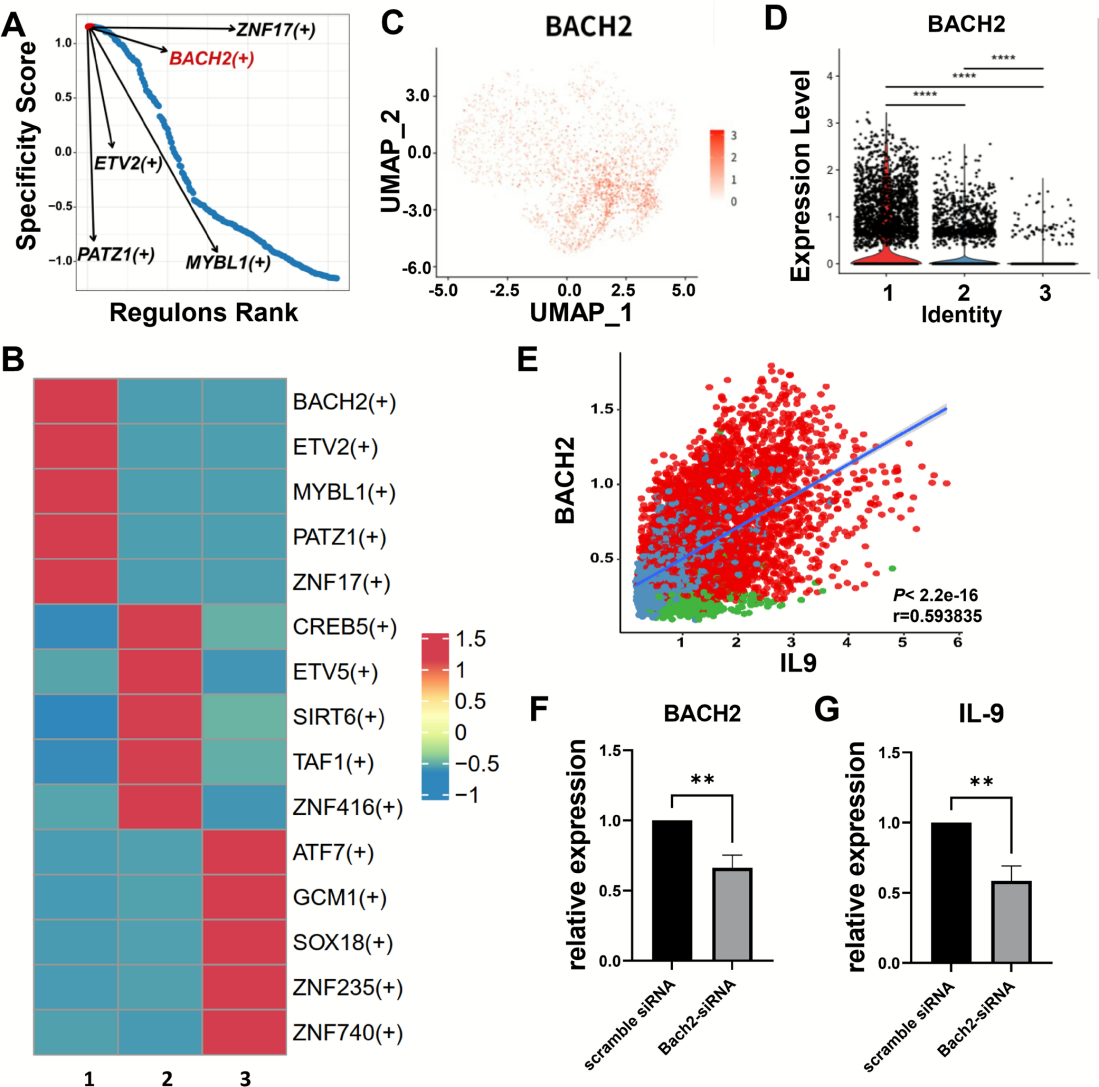
Bach2 is the specific transcription factor of ILC9s. (A) Rank for regulons in Cluster 1 based on regulon specificity score (RSS). (B) Heatmap showing TF activity for each cluster. (C) The UMAP visualization of BACH2. (D) Expression distribution (violin plots) of BACH2 in each cell cluster. (E) The correlation of BACH2 with IL‐9. (F, G) The relative expression of Bach2 and IL‐9 mRNA. Data are shown as mean ± SEM. **p < 0.01.

To further investigate the transcription factor of ILC9, we applied the SCENIC pipeline to analyze the scRNA‐seq data. Our network analysis identified Bach2 as the most specific regulon associated with cells in Cluster 1, signified that Bach2's activity was more concentrated in cluster 1 (Figure 4A). Importantly, we showed the heatmap of transcription factor activity for each cluster that Cluster 1 exhibited much higher activity of transcription factor Bach2 compared to the other clusters (Figure 4B). Then, the UMAP and violin plots provide additional support that the expressions of this regulon are highly specific to Cluster 1 (Figure 4C,D). Different from Th9, PU.1 and IRF4 which are essential for the development of Th9 did not show high activity and expression in ILC9 cluster (Figures S3D and S5B). Bach2 was reported to be associated with the inhibition of Th2 cytokine production and Th2 cell differentiation [42]. However, the link between Bach2 and IL‐9 is rarely reported. Then, we found a moderate positive correlation between the proportion of IL‐9^+^ILCs and the proportion of Bach2^+^ILCs among the overall ILC populations (Figure 4E).

These analyses indicated that the transcription factor Bach2 might be the candidate for ILC9 fate master. The prediction provided a guide for our further experimental investigation. Next, we examined whether the transcription factor Bach2 regulates the expression of IL‐9. We treated freshly isolated PBMCs with IL‐4 and TGF‐β and, followed by the transfection of Bach2 siRNA. Figure 4F shows the efficient decrease of Bach2 mRNA. We found a significant decrease in IL‐9 mRNA expression after the knockdown of Bach2 (*p <* 0.01, Figure 4G). Together, these findings suggest that Bach2 contributes to IL‐9 regulation in cytokine‐stimulated PBMCs and represents a candidate transcription factor for ILC9s.

### 3.5 ILC9s Are Highly Enriched in H1R ^+^ OX‐40 L^−^ ILCs

**FIGURE 5:**
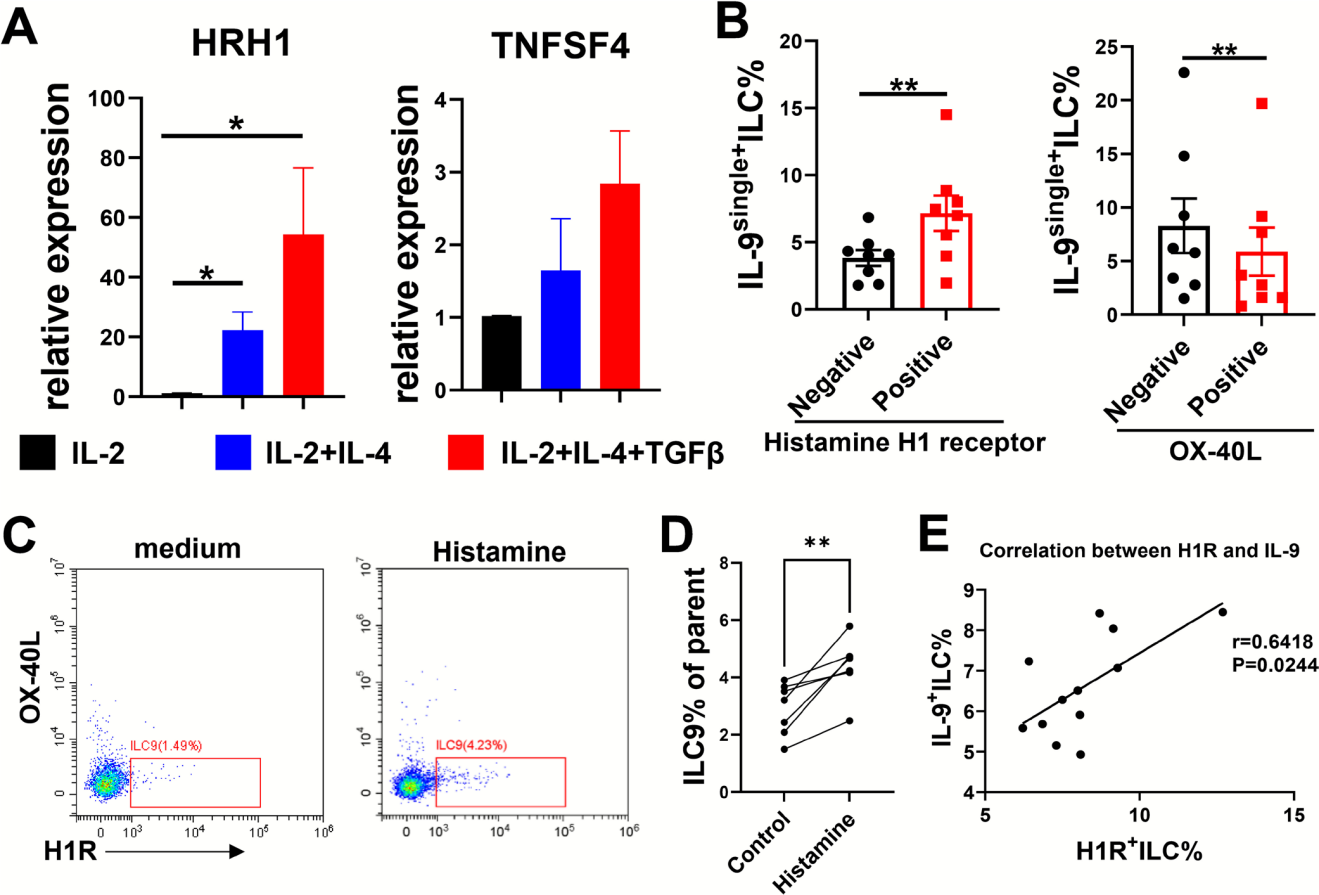
ILC9s are highly enriched in H1R^+^OX‐40L^−^ILCs. Human ILCs were treated with IL‐2, IL‐4, and TGF‐β for 7 days. (A) The bar graphs of HRH1 and TNFSF4 mRNA levels in indicated groups. (B) The percentages of IL‐9 single^+^ILC in Histamine H1 receptor and OX‐40L‐positive and negative ILCs were determined by flow cytometry (“IL‐9single^+^” = IL‐9^+^/IFN‐γ^−^/IL‐13^−^/IL‐17A^−^). Human PBMCs were treated with histamine for 24 h. (C) The flow plots of ILC9. (D) The dot graphs of ILC9%. (E) The correlation of HRH1^+^ILC with IL‐9^+^ILC. Data are shown as mean ± SEM. *p < 0.05 and **p < 0.01.

To further determine the profile of ILC9, we assessed the expressions of *HRH1* and *TNFSF4* under the aforementioned conditions in vitro. Consistently, we found IL‐4 alone promoted the levels of *HRH1* in ILCs (*p <* 0.05), and the addition of TGF‐β further enhanced the expression of *HRH1* (*p <* 0.05, Figure 5A). However, the levels of *TNFSF4* were equivalent among all stimuli (Figure 5A). Using flow cytometry, we sought to determine the ILC9 phenotype (“IL‐9single^+^” = IL‐9^+^/IFN‐γ^−^/IL‐13^−^/IL‐17A^−^) after activation. ILC9s were significantly enriched in H1R^+^ and OX‐40L^−^ ILCs, respectively (Figure 5B). These data also suggested that H1R^+^OX‐40L^−^ILCs contained most IL‐9^single+^ cells. For the alleviation of allergic symptoms, H1R antagonists are the second most frequently prescribed therapeutic drugs in AR, such as cetirizine, loratadine, and desloratadine. Given that, we assumed that ILC9 might be a target of these therapeutic drugs via H1R. Thus, we treated PBMCs from patients with AR with histamine and their purified ILCs with H1R or H2R agonists in vitro. We found significant up‐regulation of ILC9 percentages in the PBMCs from patients with AR under the treatment of histamine (*p <* 0.01, Figure 5C,D). The levels of IL‐9 and HRH1 didn't exhibit any statistical difference under the treatment of either H1R (2‐Pyridylethylamine dihydrochloride, 10 μM) or H2R (Amthamine dihydrobromide, 10 μM) agonists (Figure S3F). These might because that histamine engaged a broader or different set of pathways than the agonists which affected the development of ILC9s. However, we found that the percentages of IL‐9^+^ILCs were significantly positively correlated with H1R^+^ILCs (*p* = 0.0244, Figure 5E).

Taken together, these data demonstrate that IL‐9 was mainly produced by H1R^+^OX‐40L^−^ILCs. Hence, we suggest tentatively naming the H1R^+^OX‐40L^−^ILCs as ILC9s in the present study.

### 3.6 Patients With AR Expressed Higher Levels of ILC9s That Are Suppressed by Subcutaneous Allergen‐Specific Immunotherapy and PPARγ Antagonist

**FIGURE 6:**
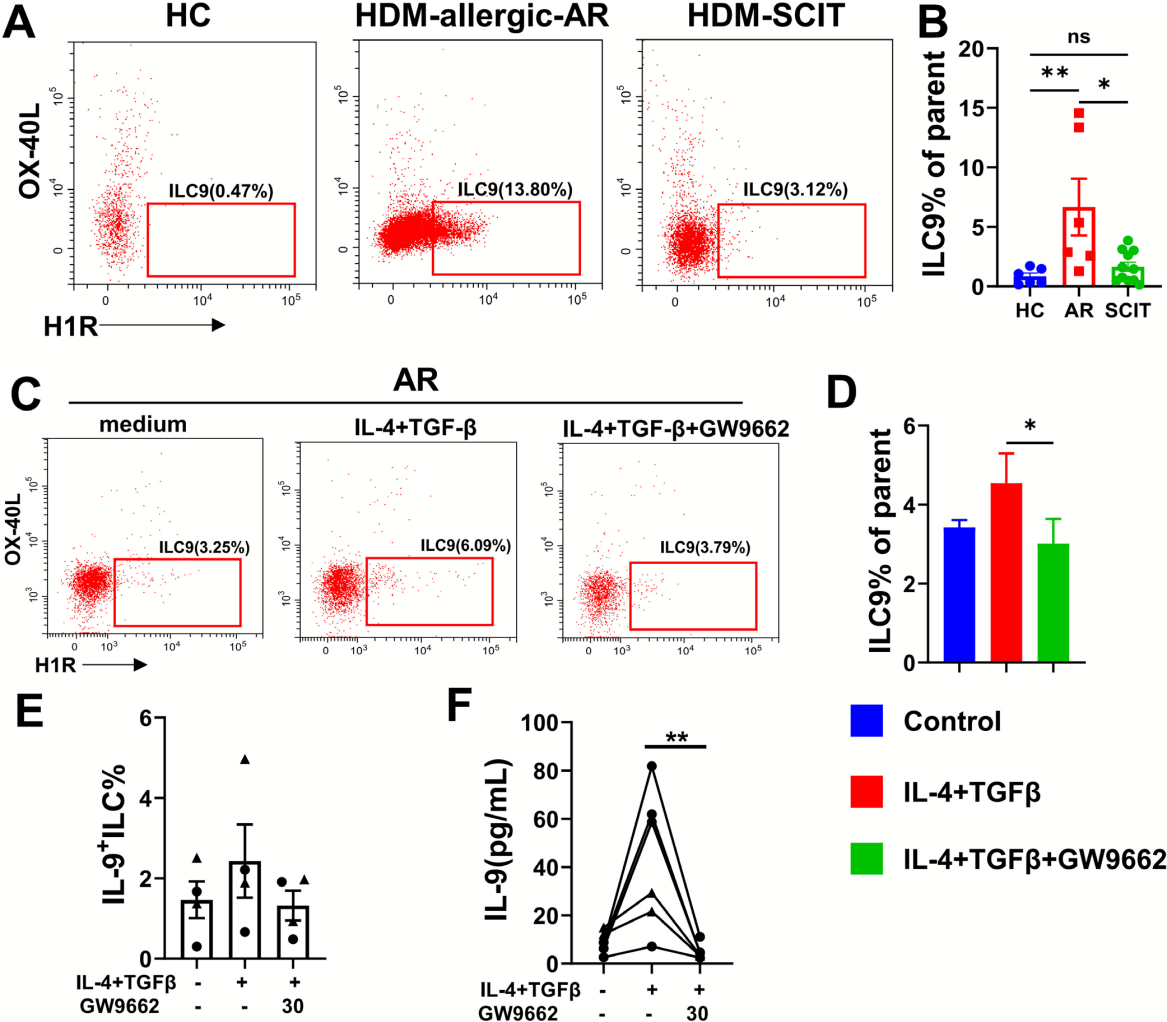
Patients with AR expressed higher levels of ILC9s that are suppressed by subcutaneous allergen‐specific immunotherapy and PPARγ antagonist. (A) The flow plots of ILC9 from healthy donors, patients with AR or patients undergoing subcutaneous immunotherapy (SCIT) (*n* = 6–10). (B) The bar graphs of ILC9%. (C, D) The levels of ILC9 after indicated stimulus in the presence or absence of PPARγ antagonist GW9662 (30 μM) for 3 days. (E) The levels of IL‐9 in the supernatants. (F) The percentages of IL‐9 + ILC. Data are shown as mean ± SEM. *p < 0.05 and **p < 0.01.

Our findings that ILC9s displayed high levels of H1R led us to consider that the ILC9 may play a vital role in the pathogenesis of AR. Therefore, we compared healthy subjects with patients with HDM‐induced AR and observed a marked increase in the percentage of ILC9s (*p <* 0.01, Figure 6A,B). The gating strategy was shown in Figure S2B,C. These studies aimed to demonstrate that ILC9s appeared to be associated with the development of AR. Subcutaneous immunotherapy (SCIT) is increasingly considered a well‐validated and only potentially immune‐modifying and curative treatment for patients with HDM‐induced AR. ILC2s have recently emerged to play an important role in the development of allergic inflammation. As ILCs are functionally plastic, we enrolled patients with HDM‐induced AR and receiving SCIT for at least 6 months to determine if there was ILC phenotype shifting in responder SCIT patients. The clinical and demographic parameters were displayed in Table 1. There were comparable levels of IgE concentration, TNSS, and VAS score between HDM‐induced AR and responder SCIT patients and were both lower than those of healthy subjects. Subjects with HDM‐induced AR revealed a significantly higher frequency of ILC9s in PBMCs compared to responder SCIT patients (*p <* 0.05, Figure 6A,B). Meanwhile, we observed that the responder SCIT patients had a comparable proportion of ILC9s to healthy subjects (Figure 6B). It indicates that SLIT treatment is effective in down‐regulating the levels of ILC9 levels.

As aforementioned, we found an up‐regulation of PPARγ in ILCs, which coincided with an increase in the synthesis of IL‐9 following the stimulation of IL‐4 and TGF‐β for 7 days in vitro. Thus, we questioned whether PPARγ is associated with the IL‐9 differentiation pathway. To address this, we pre‐treated the PBMCs from patients with AR with the PPARγ antagonist, GW9662, followed by the stimulation of IL‐4 and TGF‐β. We found that the treatment with GW9662 could reverse the elevated percentages of ILC9 (*p <* 0.05, Figure 6C,D) and IL‐9 protein levels (*p* < 0.01, Figure 6F) by IL‐4 and TGF‐β. However, the percentage of IL‐9^+^ILC remained unchanged (Figure 6E). Taken together, these data indicate that allergic individuals exhibit a higher expression of ILC9s than healthy controls. This might be a possible mechanism that explains who is a responder to SCIT with shifted circulating IL‐9‐producing ILC9s to other subgroups. Importantly, IL‐4 and TGF‐β induced ILC9 differentiation, which could be suppressed by the PPARγ antagonist. These findings reveal the presence of ILC9s in patients with AR and the involvement of PPARγ in the differentiation of ILC9.

## 4 Discussion

It is estimated that 8%–10% of the global population suffers from one or more allergic diseases, including mild rhinitis, asthma, food allergy, and AD [22, 43, 44]. The interplay between ILC2s and immune cells might be an important factor contributing to the pathogenesis of chronic skin or mucosal inflammatory diseases, such as AD and asthma [1, 2, 25, 45]. Given the high expression of IL‐9 in the nasal mucosa of patients with AR, we defined a new subgroup of ILC, namely ILC9, by sc‐RNAseq for the first time. We demonstrated that the levels of H1R^+^OX‐40L^−^ILC9s were up‐regulated in patients with AR, and the responder SCIT patients displayed a comparable proportion of ILC9s to healthy subjects. This study sheds new light on the characteristics of a new subgroup of ILCs, namely the ILC9 subset.

It's increasingly acknowledged that ILCs are functionally plastic and quickly adapt to microenvironment changes to promote distinct types of immune response. However, previous studies mostly focus on the type 2 immune cytokines expression regarding allergic diseases in the blood, sputum, or sometimes in the tissues. Nouri‐Aria et al. [46] and our study provided the increase of IL‐9 in the nasal mucosa of patients with AR by immunofluorescence staining. IL‐9 is a pleiotropic cytokine that could be produced by a wide variety of cells, including mast cells, T cells, and ILC2 [3, 47, 48]. To our knowledge, it's the first report demonstrating the in vivo presence of IL‐9 in lineage‐negative ILCs in the human nasal mucosa of allergic patients. Whether ILC9 constitutes an epigenetically and functionally distinct subset of ILCs is a contentious issue. Here, we showed the existence of IL‐9‐producing ILCs without the expression of the ILC2 transcription factor GATA3. These data suggested that there might be a unique subset of ILCs mainly producing IL‐9.

Characterization of ILC9s strongly depended on the primed environment of the cells. Circulating ILCs, in response to IL‐4 and TGF‐β, differentiate into stable IL‐9‐producing mature ILC9s. Hence, we speculated that ILCs possess a functional plasticity dependent on the preconditioning by tissue‐derived factors. TGF‐β is highly localized in the area underlying the epithelial branching point and evokes airway hyperresponsiveness in asthma [49]. IL‐9 is produced by the ILCs differentiated in the presence of TGF‐β plus IL‐4, suggesting that TGF‐β and IL‐4 together are inducing a new gene program partially by PPAR‐γ. Our results identified that PPAR‐γ was preferentially expressed by IL‐9^+^ILCs, and functional analysis revealed that a PPAR‐γ antagonist could suppress the production of ILC9s in cells isolated from patients with AR. Consistent with our findings, PPAR‐γ has recently been shown to function in lung tissue rather than in the lung‐draining medLNs [50]. We demonstrate in the present study that TGF‐β can induce high levels of PPAR‐γ in ILCs and thus uncover a putative differentiation step toward ILC9s. Together, our study presumed that TGF‐β might represent a central mediator of ILC9s via the induction of PPAR‐γ.

Our scRNA‐seq analysis revealed that a proportion of circulating H1R^+^OX‐40 L^−^ ILCs express high levels of IL‐9 rather than canonical IL‐5 and IL‐4 and specific regulon Bach2. We found the H1R^+^OX‐40L^−^ILCs contained most IL‐9^single+^ cells, without the secretion of IFN‐γ, IL‐13, and IL‐17A, which separate ILC9 from other ILC subtypes. ILC2s are known to produce both IL‐5 and IL‐13 upon activation. Some studies indicated that IL‐5 transcripts peak earlier and then decrease, while IL‐13 transcripts continue to increase over time after stimulation, suggesting a temporal difference in production. Given the long duration of culture, we chose IL‐13 instead of IL‐5 as the cytokine marker of ILC2.

It has been reported that Bach2 cooperating with BATF binds to AP‐1 motifs and inhibits Th2 cytokine production [42], meanwhile, it induces the IL‐9 production [20]. Bach2 knockdown led to decreased IL‐9 levels, supporting a role for Bach2 in IL‐9 regulation. It may serve as a candidate transcription factor for ILC9s, although further studies using purified ILC populations will be required to validate its specificity. Unlike Th9 cells that depend on PU.1 and IRF4 [13, 16], ILC9s did not show specific upregulation of these transcription factors, suggesting distinct regulatory pathways controlling IL‐9 expression in ILCs. However, due to the limitation of 10× genomic technology difficult in capturing and analyzing cytokine expression accurately, we added the cytokine stimulus and PMA/ionomycin inducing cells into a highly activated state the same as used for flow cytometry analyses. Nevertheless, the addition of PMA/ionomycin could not capture the full spectrum of naturally occurring cellular diversity; this was the disadvantage of the usage of a strong stimulation.

Histamine plays essential roles in the induction of allergic inflammation by activating smooth muscle cells, epithelial cells, granulocytes, dendritic cells, and macrophages, as well as T cells [51]. In T cells, histamine H4‐receptor signaling has been shown to increase IL‐9 production in Th9‐polarized cells [52]. Here, we found that histamine could also promote the differentiation of ILC9 most likely via H1R. H1R antagonists have long been used to treat allergic diseases with satisfactory outcomes. Our observations showed a significant positive correlation between IL‐9 and H1R levels in ILCs. However, no difference was found in their expressions under the stimulations of selective H1R and H2R agonists. These might be because histamine engaged a broader or different set of pathways than the agonists which affected the development of ILC9s.

The H1R antagonism for the treatment of allergic diseases, although effective in allergic rhinitis, has its limitations. Loratadine has been used to temporarily relieve and prevent the symptoms of AR and other allergies, whereas it shows fewer benefits in the treatment of allergic asthma. To date, SCIT is considered the only potentially immune‐modifying and curative treatment for allergic patients, including the comorbidity of AR and asthma. In a prospective, double‐blind, placebo‐controlled trial, the proportion of IL‐10‐producing ILC2 was restored from the classical type 2 IL‐5^+^IL‐13^+^ILC2 in patients who received grass‐pollen sublingual immunotherapy in parallel to healthy subjects [24]. Similarly, the ratio of ILC1/ILC2 proportion in the PBMCs of HDM‐AIT responders was increased compared to those in the nonresponders [53]. Together, these results proposed that although ILCs lack the antigen‐specific receptors, their phenotype shifting in AIT is obvious. Here, we demonstrated that HDM SCIT attenuated the numbers of IL‐9‐producing ILCs close to those in healthy subjects. Our observations that ILC9 proportions were down‐regulated in HDM‐induced AR patients receiving SCIT suggested their potential as a biomarker of patient responsiveness.

We acknowledge that there are some limitations to this study. Although we showed that the characteristics of ILC9s, the specificity of their embryology and epigenetic inheritance should be further determined as a distinct subset. Another limitation is the cross‐sectional design of this study and the lack of comparison with nonresponder patients, which will be addressed in future longitudinal studies. A follow‐up study is being planned to address this issue.

In conclusion, we identified a plastic functional subset of IL‐9 producing ILC that has the surface phenotype of H1R^+^OX‐40L^−^ILC9 in the present study. The increased frequencies of ILC9s in patients with HDM‐AR were reversed in HDM‐SCIT responders. This study warrants further investigations to validate the use of ILC9s as a biomarker of AIT response.

## Author Contributions

Y.‐Q.P. had primary responsibility for the framework of the study, data analysis, and manuscript preparation. Y.‐Q.P., T.Z., X.‐Q.L., I.O., Q.S., B.R., B.‐X.H., D.‐H.C., H.M., H.‐J.Y., Q.‐L.F., and C.A.A. contributed to the conception and design of the study and interpretation of results; Q.‐L.F. and C.A.A. supervised the project. Y.‐Q.P., T.Z., X.‐Q.L., I.O., B.R., B.‐X.H. performed the experiments. Q.S. and Y.‐Q.P. conducted data analysis and interpretation of the results.

## Funding

This study was supported by grants from NSFC (82201248, 82571286, 82271144, 81970863 and 82401310), Guangzhou Key R&D Program (2023B03J1233), and NSFC Incubation Project of Guangdong Provincial People's Hospital (KY0120220032).

## Ethics Statement

The study was approved by the Ethics Committee of The First Affiliated Hospital, Sun Yat‐sen University, China (No. [2017]138). Signed informed consent and required documentation were obtained from each patient and respective guardian before the study. Human blood buffy coats from “anonymous donors” were from Guangzhou Blood Center; the exemption of written informed consent was approved.

## Conflicts of Interest

The authors declare no conflicts of interest.

## Supporting information


**Figure S1:** The existence of ILC9 in human tonsils and nasal mucosa of patients with AR. (A) Immunofluorescence staining of human tonsil tissue from nonallergic donors, showing ILC9s by staining for Lineage (including CD3, CD20, FcɛRI, gray), IL‐9 (red), GATA 3 (green), and DAPI (blue). (B, C), Immunofluorescence staining of nasal mucosa of patients with AR, showing ILC9s by staining for Lineage (including CD3, CD20, FcɛRI, green), IL‐9 (red), IL‐5/13(cyan), and DAPI (blue).
**Figure S2:** Sorting strategy of ILCs and control staining for ILC9s. (A) Human blood ILCs were sorted as Live^+^CD45^+^Lin^−^CD127^+^ cells. (B) The purity of expanded ILCs. (C) The blank control for ILC9s. (D) PBMCs from patients with AR were staining with ILC markers only, which served as FMO staining control for ILC9s.
**Figure S3:** IL‐9^+^ILC priming condition. (A) Indicated dose of TGF‐β, EGF, AREG and time points were tested to determine the optimal condition for IL‐9‐priming. The graphs of IL‐9^+^ILC% were shown. (B) Human ILCs were cultured in the presence of IL‐2 (100 U/mL), IL‐2 plus IL‐4 (20 ng/mL), or all together with TGF‐β (3 ng/mL) for 7 days. The levels of IFN‐γ, IL‐4, IL‐5, IL‐17A/F, and IL‐10 in the supernatants. (C) The bar graphs of IL‐13, IL‐17A, IFN‐γ mRNA levels and GATA3, RORγt, T‐bet mRNA, and MFI levels. (D) The bar graphs of PU.1 mRNA levels. Data are shown as mean ± SEM. *p < 0.05.
**Figure S4:** scRNA‐seq analysis of human ILCs in peripheral blood. Human ILCs were treated with IL‐2, IL‐4 and TGF‐β for 7 days and then re‐stimulated with PMA and Ion for 4 h before scRNAseq. (A) UMAP visualization of scRNAseq data of all ILCs sequenced. (B) Differential gene expression analysis between clusters are presented as heatmap. (C) The expression of IL‐9. (D) Expression distribution (violin plots) of IL‐9, IL‐5, and IL‐4 in the new clustering. (E) Differential gene expression analysis between cluster 0 and cluster 1 are presented as a volcano plot. (F) The levels of IL‐9 and HRH1 with the treatment of H1R agonist (10 μM) or H2R agonist (10 μM).
**Figure S5:** UMAP visualization of co‐expression data. (A) The co‐expression of IL‐9 with IL‐5, IL‐17A, IFN‐γ, and IL‐10. (B) UMAP visualization and violin plot of IRF4.

**Table S1:** The primer sequences used for qRT‐PCR.

## Data Availability

The data that support the findings of this study are available by reasonable request at OMIX with accession number: OMIX012222. This paper does not report original code.
