# Supplementary material for: Characterization of Human Group 9 Innate Lymphoid Cells in Response to Allergen Immunotherapy in Patients With Allergic Rhinitis
Source: Allergy. 2025 Dec 26;81(5):1650–64. doi: 10.1111/all.70202 (PMC13139807; doi:10.1111/all.70202)

Supplementary Figure 1

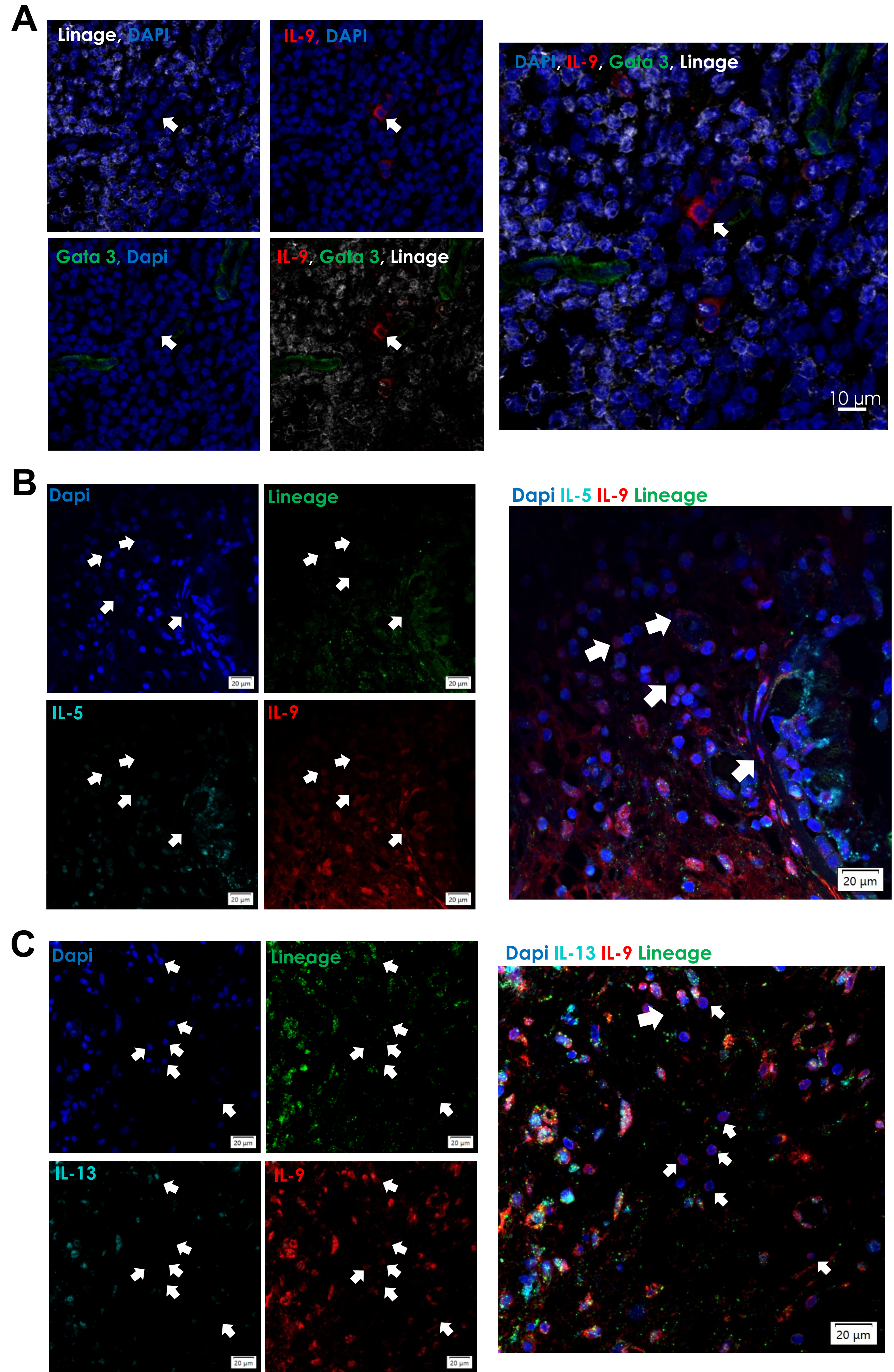

Supplementary Figure 2

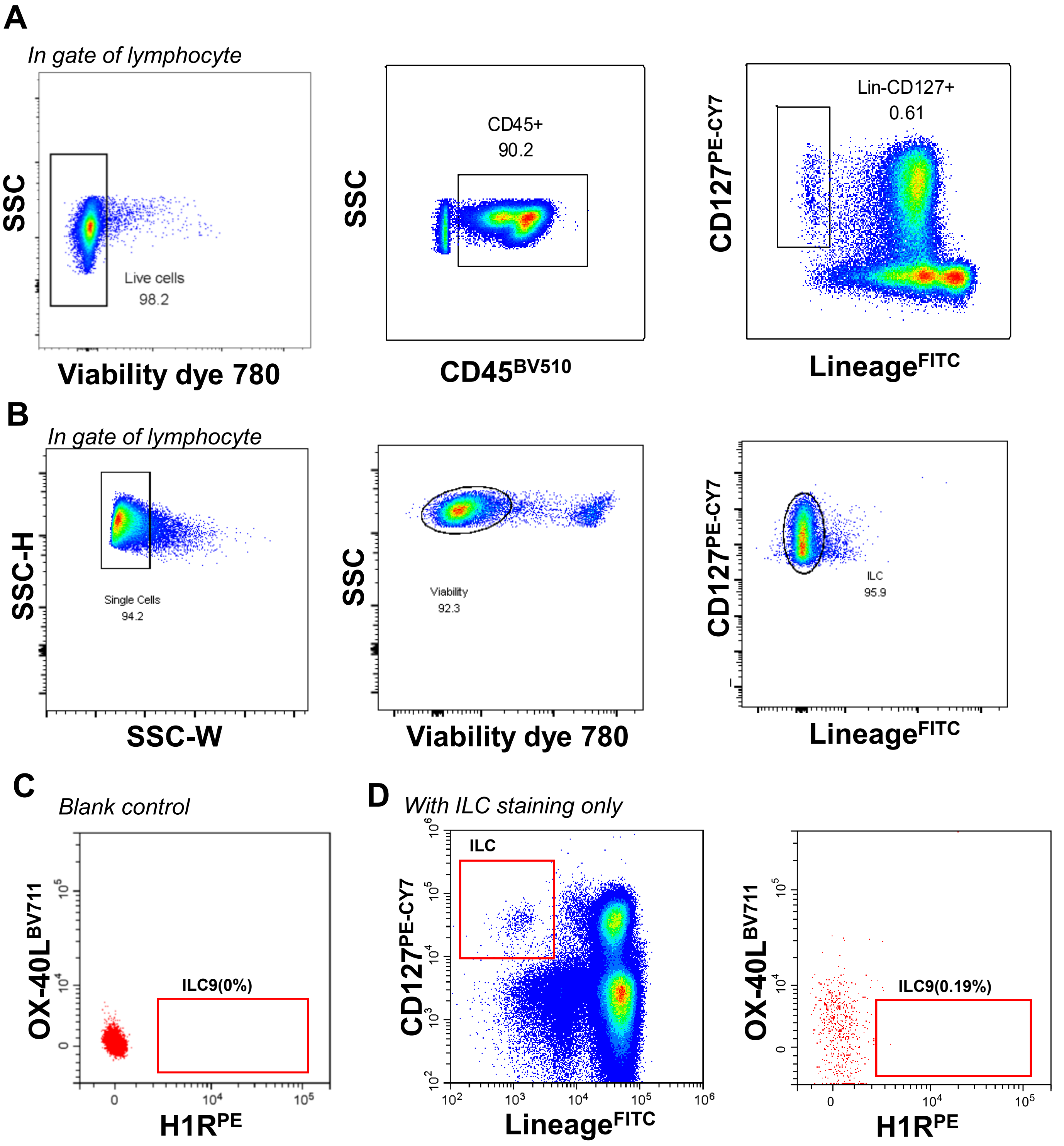

Supplementary Figure 3

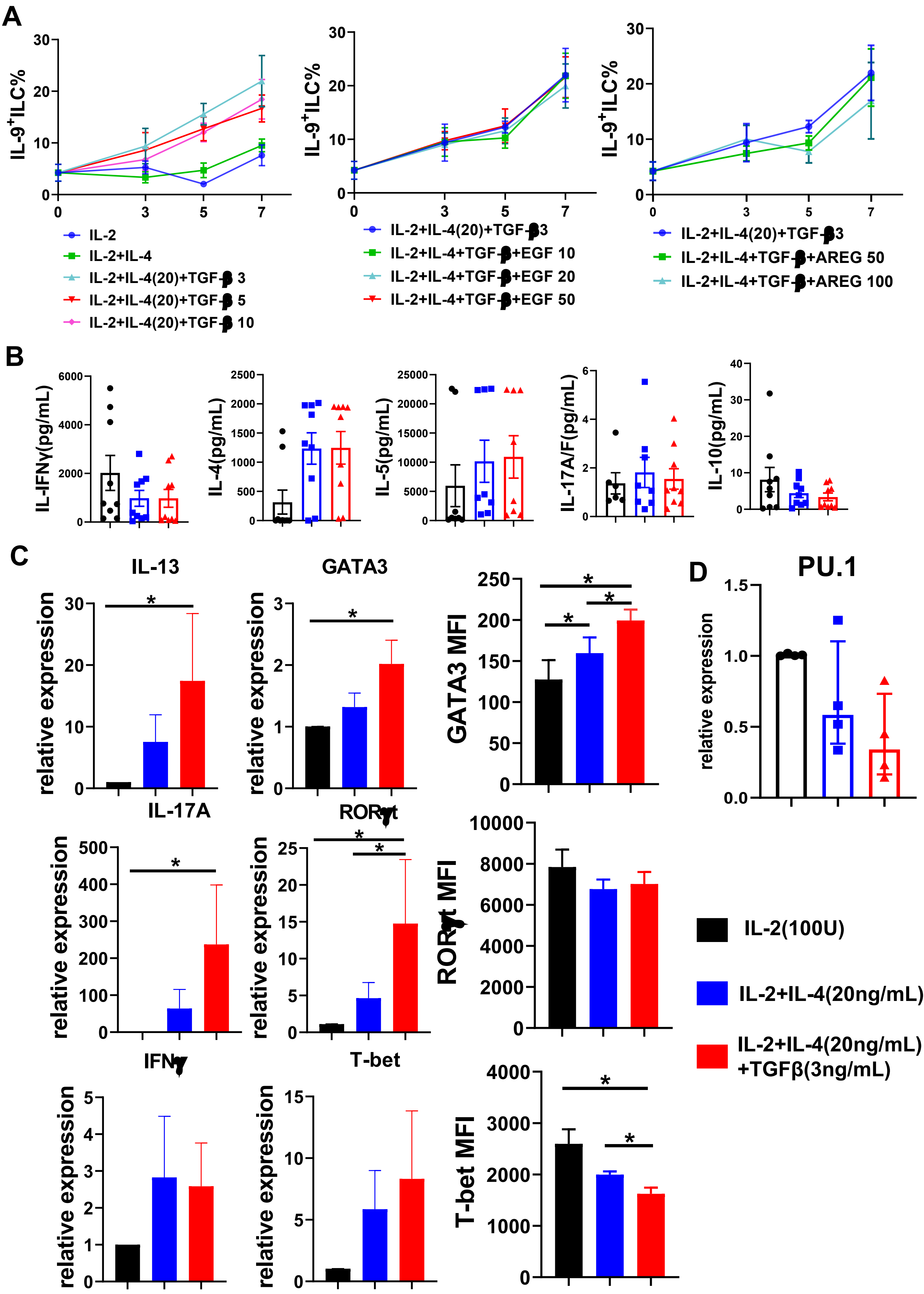

Supplementary Figure 4

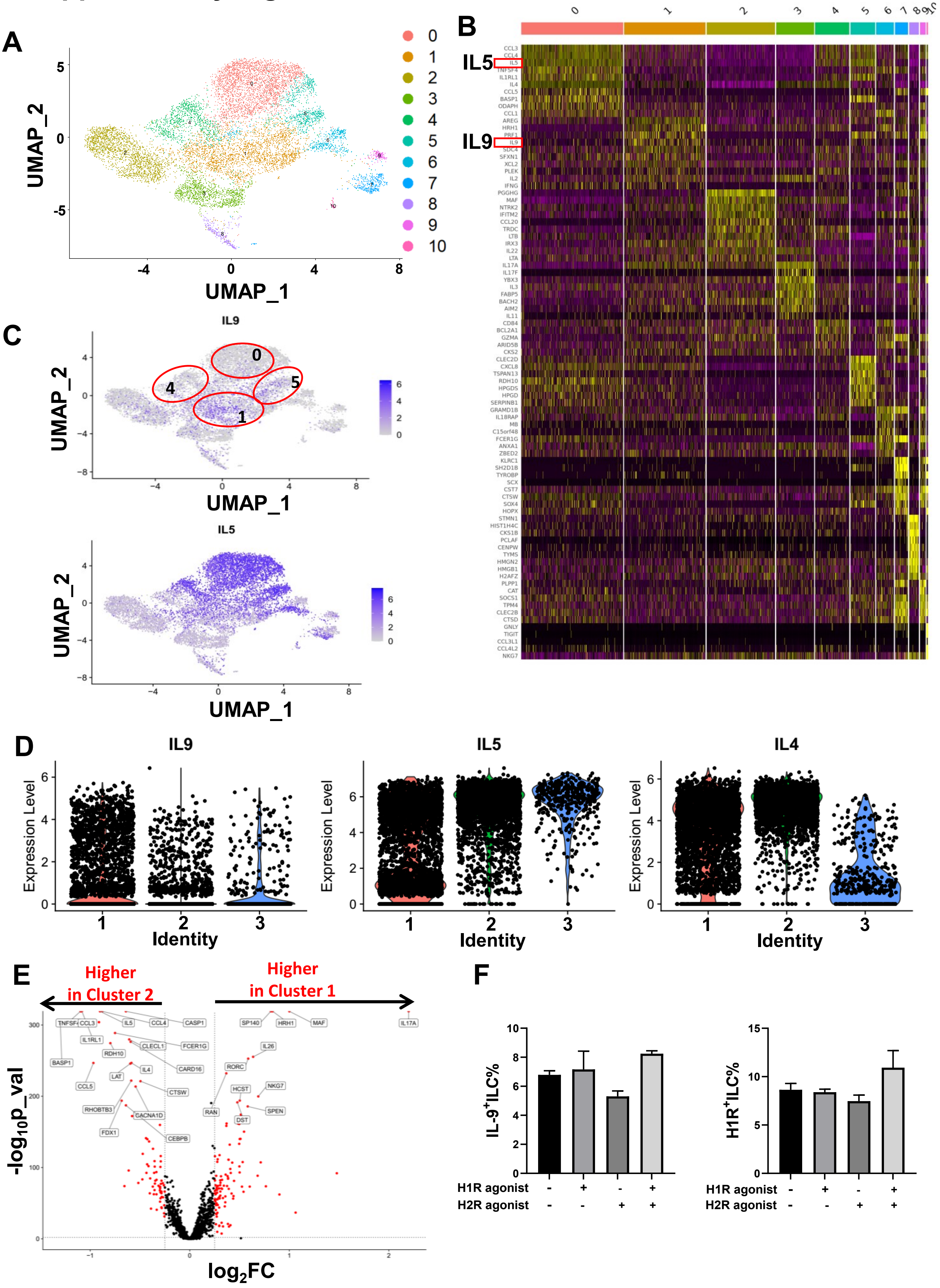

Supplementary Figure 5

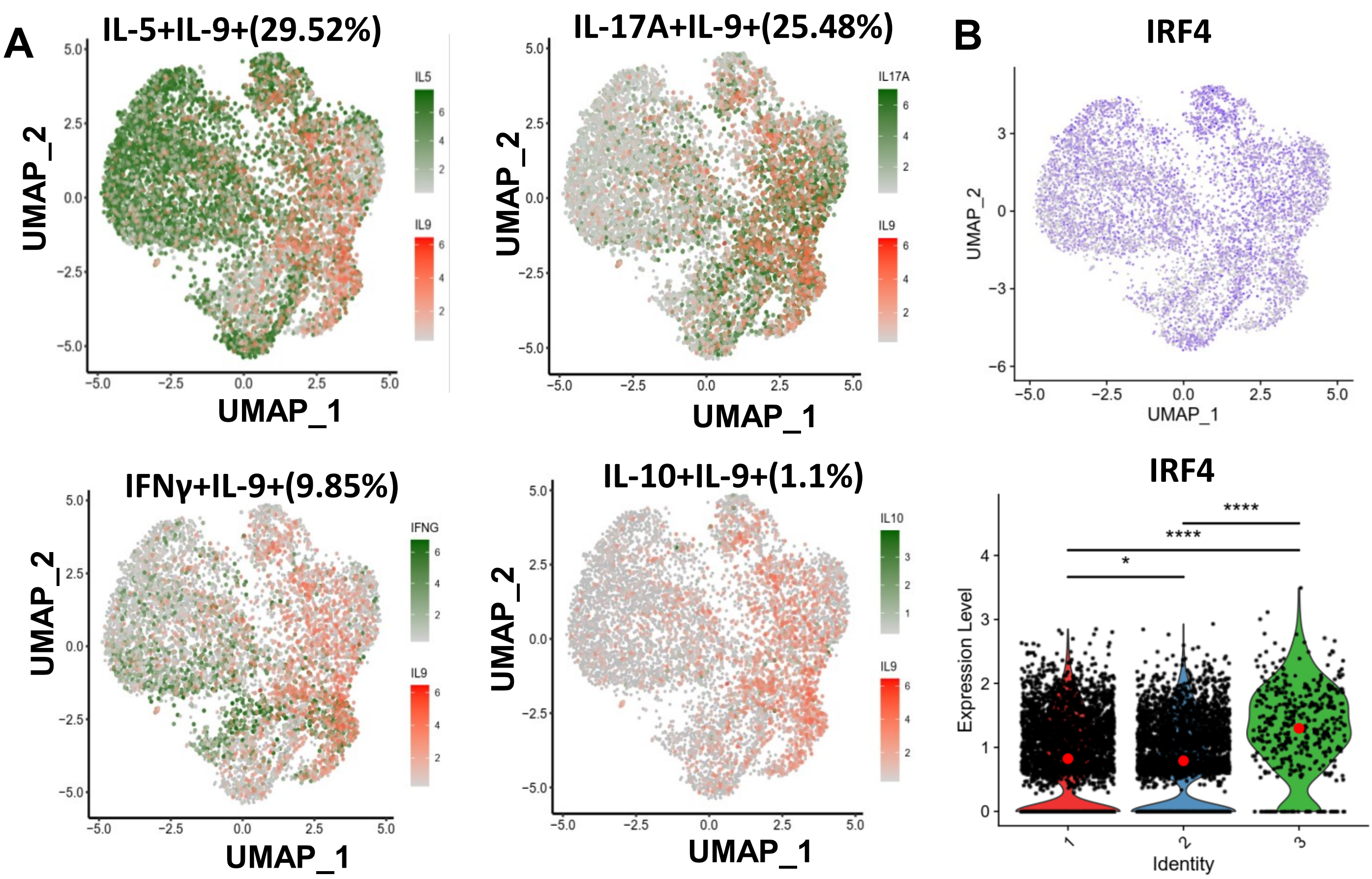

Supplement: Supplementary file 1 — Figure S1: The existence of ILC9 in human tonsils and nasal mucosa of patients with AR. (A) Immunofluorescence staining of human tonsil tissue from nonallergic donors, showing ILC9s by staining for Lineage (including CD3, CD20, FcɛRI, gray), IL‐9 (red), GATA 3 (green), and DAPI (blue). (B, C), Immunofluorescence staining of nasal mucosa of patients with AR, showing ILC9s by staining for Lineage (including CD3, CD20, FcɛRI, green), IL‐9 (red), IL‐5/13(cyan), and DAPI (blue). Figure S2: Sorting strategy of ILCs and control staining for ILC9s. (A) Human blood ILCs were sorted as Live+CD45+Lin−CD127+ cells. (B) The purity of expanded ILCs. (C) The blank control for ILC9s. (D) PBMCs from patients with AR were staining with ILC markers only, which served as FMO staining control for ILC9s. Figure S3: IL‐9+ILC priming condition. (A) Indicated dose of TGF‐β, EGF, AREG and time points were tested to determine the optimal condition for IL‐9‐priming. The graphs of IL‐9+ILC% were shown. (B) Human ILCs were cultured in the presence of IL‐2 (100 U/mL), IL‐2 plus IL‐4 (20 ng/mL), or all together with TGF‐β (3 ng/mL) for 7 days. The levels of IFN‐γ, IL‐4, IL‐5, IL‐17A/F, and IL‐10 in the supernatants. (C) The bar graphs of IL‐13, IL‐17A, IFN‐γ mRNA levels and GATA3, RORγt, T‐bet mRNA, and MFI levels. (D) The bar graphs of PU.1 mRNA levels. Data are shown as mean ± SEM. *p < 0.05. Figure S4: scRNA‐seq analysis of human ILCs in peripheral blood. Human ILCs were treated with IL‐2, IL‐4 and TGF‐β for 7 days and then re‐stimulated with PMA and Ion for 4 h before scRNAseq. (A) UMAP visualization of scRNAseq data of all ILCs sequenced. (B) Differential gene expression analysis between clusters are presented as heatmap. (C) The expression of IL‐9. (D) Expression distribution (violin plots) of IL‐9, IL‐5, and IL‐4 in the new clustering. (E) Differential gene expression analysis between cluster 0 and cluster 1 are presented as a volcano plot. (F) The levels of IL‐9 and HRH1 with the treatment [file ALL-81-1650-s001.pdf]
