# Supplementary material for: Characterization of Human Group 9 Innate Lymphoid Cells in Response to Allergen Immunotherapy in Patients With Allergic Rhinitis
Source: Allergy. 2025 Dec 26;81(5):1650–64. doi: 10.1111/all.70202 (PMC13139807; doi:10.1111/all.70202)
Supplement: Supplementary file 2 — Table S1: The primer sequences used for qRT‐PCR. [file ALL-81-1650-s002.docx]

| **Primers** | | **（5’-）** |
| --- | --- | --- |
| **GAPDH** | sense | GAGTCAACGGATTTGGTCGT |
|  | antisense | TTGATTTTGGAGGGATCTCG |
| **BACH2** | sense | AAGGTCTCTGTTCAGCATAACG |
|  | antisense | CCTGAGTGATCCCCTTGTCAAAA |
| **HRH1** | sense | CTGAGCACTATCTGCTTGGTC |
|  | antisense | AGGATGTTCATAGGCATGACGA |
| **TNFSF4** | sense | GGTCAGGTCTGTCAACTCCTT |
|  | antisense | CATCCAGGGAGGTATTGTCAGT |
| **GATA3** | sense | AAAGAAGGCATCCAGACCCG |
|  | antisense | TTGAAGGAGCTGCTCTTGGG |
| **RORγt** | sense | TCTACACGGCCCTGGTTCTCATCAA |
|  | antisense | GGAAGGCGGCTTGGACCACGAT |
| **IFNγ** | sense | TCGGTAACTGACTTGAATGTCCA |
|  | antisense | TCGCTTCCCTGTTTTAGCTGC |
| **T-bet** | sense | CAGGGACGGCGGATGTTC |
|  | antisense | CTTTCCACACTGCACCCACT |

**Supplementary Table：**The primer sequences used for qRT-PCR.
